# Cross-cultural validation of the Child Adolescent Teasing Scale for Colombian students

**DOI:** 10.1590/1518-8345.2099.2968

**Published:** 2018-05-17

**Authors:** Karol Johanna Briñez Ariza, Clara Virginia Caro Castillo, María Elena Echevarría-Guanilo, Marta Lenise Do Prado, Silvana Silveira Kempfer

**Affiliations:** 1 MSc, Nursing. Doctor Degree Student, Nursing, Universidad Nacional de Colombia, Bogotá, Bog, Colombia. Researcher. Doctor Degree Sponsorship: Beca Colciencias Colombia para el Doctorado en Enfermeria.; 2 PhD. Full Professor, Nursing, Universidad Nacional de Colombia, Bogotá, Bog, Colombia.; 3 PhD, Nursing. Professor, Nursing, Universidad Federal de Santa Catarina, Florianopolis, SC, Brazil.; 4 PhD, Nursing Philosophy. Full Professor, Nursing, Universidad Federal de Santa Catarina, Florianopolis, SC, Brazil.; 5 PhD, Nursing. Professor, Nursing, Universidad Federal de Santa Catarina, Florianopolis, SC, Brazil.

**Keywords:** Validation Studies, Surveys and Questionnaires, School Nursing, Nursing, Nursing Methodology Research

## Abstract

**Objective:**

to carry out the cross-cultural validation of the instrument “Child Adolescent Teasing Scale” for the Colombian student population.

**Method:**

methodological study carried out with students aged 8 to 15, from public and private educational institutions in the municipality of Ibagué, Colombia. The form for the characterization of students and the Child Adolescent Teasing Scale were used.

**Results:**

the cross-cultural adaptation process was organized in seven steps: comparison of the Spanish version of the instrument with the original English version, back-translation, consensus version, face validity and terminology adjustment by students, face and content validity by experts, assessment committee for the final version, pilot test and reliability.

**Conclusion:**

the version adapted to the Spanish spoken in Colombia of the Child Adolescent Teasing Scale (Escala de burlas para niños y adolescentes), which assesses the frequency and distress caused by teasing, showed desirable results in terms of validity and reliability.

## Introduction

Research in pediatric and adolescent nursing seeks to deepen the knowledge of phenomena related to health care by promoting studies that fill the gaps identified in the scientific literature and that aim to contribute to: new perspectives and alternatives for the resolution of problems affecting their health and welfare, as well as the welfare of their family. 

One of the most prevalent phenomena in today’s society, first described more than three decades ago, is the bullying among students. It is defined as the exposure of a student to the repetitive negative actions by another or more students, or difference in power, with the intent to cause damage or discomfort through words, physical contact or other forms, such as gestures, exclusion and defamation^1^ ^-^ ^2^.

The American National Association of School Nurses defines bullying as persistent and repetitive dynamic patterns of verbal and/or nonverbal behaviors directed by one child or more children to another, who deliberately attempt to cause physical, verbal or emotional abuse in the presence of actual or perceived difference in power^3^.

It has been associated with a number of damages and consequences for the health of children and adolescents, and has become internationally recognized as a public health problem^4^.

The problem mentioned above has negative consequences for victims, such as inability to defend themselves, powerlessness, sadness, crying, fear, loss of concentration and suicidal ideation^5^. Depression, anxiety and low self-esteem affect normal development in learning processes and their integration and adaptation in the school setting^6^. Association between bullying and different variables: Depression (p<0.01)^7^_,_ sleep problems (p<0.05), nervousness (p<0.05), restlessness (p<0.05), feelings of discomfort (p<0.05) and dizziness (p<0.05)^8^. Psychosomatic disorders in victims (p=0.0001)^9^. Physical injuries (p<0.001), symptoms of illness (p<0.001) and somatic complaints (p<0.001)^10^. Chromosomal changes in the telomeres (p=0.020), which are biomarkers of childhood stress and cell aging^11^.

According to this scenario, it is necessary to implement strategies through assessment methods for the early identification of students who experience teasing, because of their risk of becoming victims of bullying. 

There are several instruments, such as the Questionnaire for the Ombudsman’s report on school violence^12^ and the “Bullying-Cali”^13^, used for older schoolchildren.

Many of the instruments originally available were published in languages other than Spanish, which requires for the study in the Colombian context that the instruments be submitted to the processes of cultural adaptation, through the development of the translation and validation stages^14^. Thus, the instrument can be used in a different culture from which it was developed.

In this way, an instrument that could be used for students from the age of eight and that assessed the causes of teasing in all risk categories was searched, and these criteria have been met by the Child Adolescent Teasing Scale^15^. 

For the reasons described above, this study aimed to answer the question: What is the validity of the Child Adolescent Teasing Scale for the Colombian student population? It has been proposed in order to: determine the validity of the Child Adolescent Teasing Scale for the Colombian student population. 

## Method

This is a methodological study developed with students aged from 8 to 15, from public and private educational institutions in the municipality of Ibagué, Tolima (Colombia). Inclusion criteria: belong to the grades between the third grade of elementary school and the eighth grade of secondary school, and present the authorization signed by their parents.

Description of the instruments. Two instruments were applied: The form for the characterization of students and the Child Adolescent Teasing Scale. 

### Form for the characterization of students

Developed by the main author, it asked about variables such as sex, age, grade, type of educational institution; it was applied during the stages of meeting with the students: focal groups for the version by students and pilot test. 

### The Child Adolescent Teasing Scale (CATS)

It is composed of 32 items and assesses four teasing categories: physical aspect (items 11 and 21), personality and behavior (items 1, 3, 6, 7, 8, 12, 16, 20, 22, 24, 26, 27, family and home environment (items 2, 9, 10, 17, 19, 25, 29), and school-related factors (items 4, 5, 13, 14, 15, 18, 23, 28, 32). These categories are organized into two subscales: frequency and distress. The frequency subscale assesses how much the student is teased, and each item is assessed on a Likert-type scale, with the following response options: never (1) sometimes (2) often (3) and very often (4). The distress subscale assesses how much it bothers to be teased, and each item is assessed on a Likert-type scale, with the following response options: not at all (1), very little (2), more or less (3), very much (4). This scale scores values ranging from 32 to 128 for each subscale and from 64 to 256 for the full scale. The higher the value, the greater the teasing experienced by children and adolescents at school.

### Cross-cultural adaptation process

**Figure 1:**
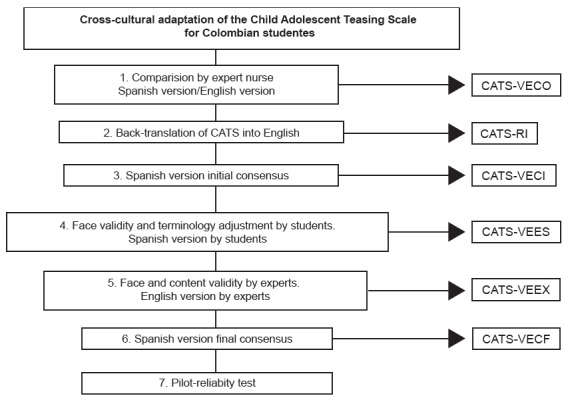
Process of cross-cultural adaptation of CATS to the Spanish spoken in Colombia, 2015-2016

The cross-cultural adaptation process was organized into seven stages^14^: Comparison of the Spanish version of CATS with the original English version, back-translation, consensus version, face validity and terminology adjustment by students, face and content validity by experts, assessment committee for the final version, pilot test and reliability (see figure 1).

Comparison of the Spanish version of CATS with the original English version. The author received the authorization, the original English version and a Spanish version, which was translated by her for Mexico and Puerto Rico. The objective was to make the necessary adjustments of CATS to the Spanish spoken in Colombia. Words and sentences were compared. A nurse with a good command of the English and Spanish languages and expertise in the validation of assessment instruments participated in this stage (CATS-VECO).

Back-translation. The objective was to translate the version resulting from the first stage into the original language of the instrument. This procedure was carried out by an English native speaker translator who back-translated it from Spanish into English (CATS-RI).

Initial consensus version (CATS-VECI). The objective was to define a version for students that included the adjustments recommended in the two previous stages (CATS-VECO and CATS-RI). It was achieved through a meeting between the supervisor of the doctoral thesis, the principal researcher and the nurse who participated in the first stage for the consensus version.

Face validity and terminology adjustment by students. The objective was to get the verbal manifestations of “how students aged 8 to 10 spoke and said those phrases” in their everyday Colombian language, so that the scale could be understood by older students. This was accomplished through a qualitative strategy according to the target population, through five focal groups and by presenting video images for each item of CATS. Opening, introductory, transitional and key questions were applied. It was concluded with refreshments served to the participants and feedback. Audio recording and transcription were performed for analysis. Changes were made and the modified version was proposed (CATS-VEES).

Face and content validity by experts. The objective was to get the assessment of CATS by six experts from Colombian Universities with experience in adapting assessment instruments, professional experience and experience in pediatric research and bullying. Each expert completed the “expert judgment” form^16^, which contained a consent for its use, and assessed five categories regarding content validity: semantic equivalence, clarity, consistency, relevance and adequacy with scores ranging from 1 (does not fulfill its function) to 4 (high level of qualification). As for face validity, the writing, precision and clarity of language of each item were evaluated, with a score of 0 (does not fulfill) and 1 (fulfill). This has allowed for suggestions that resulted in a modified version (CATS-VEEX).

Final consensus version (CATS-VECF). The objective was to make the decisions for the adjustment of the items, according to the suggestions made in the fourth and fifth stages. Consensus was reached by a committee composed of the main researcher, the supervisor of the thesis due to her professional experience with students, and a statistician who performed and interpreted the Kappa statistical test. The interpretation of the results of Kappa test was based on a theoretical reference^17^, where the strength of the Kappa coefficient is as follows: poor (0.00); slight (0.01-0.20); fair (0.21-0.40); moderate (0.41-0.60); substantial (0.61-0.80); almost perfect (0.81-1.00).

Pilot test. The objective was to check the understandability of the items and instructions for completing the instruments, calculation of completing time, need for help, and materials to make the pertinent modifications and corrections. The main researcher and the research assistant provided a CATS scale and a black pen for each student, which was positioned in a separate and individual place. For reliability, Cronbach’s alpha was calculated. Values higher than 0.50 were considered as high (possible variation from 0 to 1)^18^.

### Ethical considerations

 According to Resolution 8430 of 1993 of the Colombian Ministry of Health, this research was considered to be of minimal risk. The Ethical Committee of the Faculty of Nursing of the National University of Colombia approved the study, and authorization to perform it was obtained from the Health and Education Secretariat of Ibagué. Authorizations were requested to the parents of the students through informed consent, and to the students through an informed consent, and authorizations were also requested to the professionals to participate. 

## Results

### Comparison of the Spanish version of CATS

The original English version was compared with the Spanish version, generating a version for students (CATS-VECO). The adjustments were made in the instructions and statements that contained the words “to bother” and “to annoy”; which were replaced by “to tease”, the word most used in Colombia. The terminology was considered understandable, however, it was considered important that the instrument was submitted to face validity by Colombian students.

### Face and content validity by students

 The focal groups consisted of 42 students aged 8 to 10, from the third grade of elementary school of a public educational institution in Bogotá, with low socioeconomic status, of which 11 were female and 14 male; and of a private educational institution of Ibagué, with high socioeconomic status, of which nine were female and eight were male. 

The 32 items (CATS-VECO) were presented to the students and adjusted to their language according to their everyday speech. The proposals by the focal group were put to the vote, and during the discussion, they decided which phrase was the clearest and most appropriate. The statements of eight items were not changed because they were easily understood. All the words in the statements of eight other items had to be replaced by synonyms most commonly used in Colombia (Table 1). 

**Table 1:** Change of all 8-item words of the Spanish version of CATS*, Bogotá and Ibagué, Colombia, 2015-2016

Item #	Version items (CATS-VECI^†^)	Items changed by students (CATS-VEES^‡^)
2	My money	Being rich or poor
4	My qualifications	My grades
5	Talking too much	To talk or chat a lot in class
8	My behavior	My way of being
11	The shape of my body	Some aspect of my body
17	My jewels/chains	My ornaments or accessories
28	My school work	My school tasks
29	My progenitors	My parents

*CATS: Child Adolescent Teasing Scale.

†CATS-VECI: Spanish version initial consensus.

‡CATS-VEES: Spanish version by students.

The remaining 16 items required adjustment of the words in the statements, resulting in a different and more updated wording, or the addition of a word suggested as a synonym, or adjustments to indicate that it was related to something specific. The CATS-VEES version was proposed from this analysis (Table 2).

**Table 2:** Adjustments suggested by students of some words in the 16 statements of the items of CATS*. Bogotá and Ibagué, Colombia, 2015-2016

Item #	Version item (CATS-VECI^†^)	Items changed by students (CATS-VEES^‡^)	Item #	Version item (CATS-VECI^†^)	Items changed by students (CATS-VEES^‡^)
3	“How intelligent and prepared I am”	“How intelligent I am”;	20	“For being studious”	“For being a nerd or studious”
9	“The brand of shoes that I use”	“The brand of my shoes”	22	“For being a coward”	“For being a coward or a chicken”
10	“With whom I live”	“People that I live with”;	23	“How well I do at school”	“How well I do in the studies”
12	“Behave myself strangely or differently”	“Behave myself differently”	25	“My things”	“My things or personal items”
14	“My way of speaking”	“My way to speak”	25	“Be a fool”	“They tell me that I am a fool or a loser”
15	“Get in trouble”	“Get into a tight spot”	27	“Be shy or too quiet”	“Be shy or quiet”
16	“Behave myself as gay”	“They tell me that I am gay or lesbian”	30	“The music that I like to listen to”	“The music that I like”
19	“How my family is	“My family”	32	“Sports I participate in and do not participate in”	“Sports that I practice or play and those that I do not practice or play”

*CATS: Child Adolescent Teasing Scale.

†CATS-VECI: Spanish version initial consensus.

‡CATS-VEES: Spanish version by students.

### Validity by experts

The CATS-VECO and CATS-VEES versions were provided to the selected experts. Among the assessments, three items required clarity and wording adjustment: from “too much talking” to too much talk, “my jewels/chains” to my accessories and “get in trouble” to get into a tight spot. Three items were not clear to the experts, but to students, “Not being good at sports” “Shy or quiet” and “Having strange or different friends”. The experts approved the 16 items changed in the wording by students. 

The Kappa index showed substantial agreement for the indicators: semantic equivalence: 0.72, clarity 0.65, consistency 0.71, relevance 0.79; and moderate agreement in the adequacy indicator: 0.56. Regarding face validity, the agreement percentage among experts was calculated and showed that 84.9% of the items complied with the wording, accuracy and clarity in relation to the language. Based on these results, the CATS-VEEX version was proposed.

### Final consensus version

The necessary adjustments were made after identifying the items that were recognized by students as semantically different, but that did not alter their conceptual equivalence. In addition, the items were compared with the assessments made by experts in order to proceed the necessary adjustments of CATS, which would be used in the main study. No items were removed. The CATS-VECF version was proposed.

### Pilot test

 In this process, 19 students aged 11 to 13, from the sixth grade of elementary school of a private educational institution of Ibagué, with medium socioeconomic status, of which 6 were female and 13 male, participated. The understandability of the items and the ease of completion by students was confirmed and the time spent for completing it ranged from 10 to 15 minutes. In this stage, the reliability of the scale was calculated using the Cronbach’s alpha, with a result of 0.89 for the frequency subscale, 0.95 for the distress subscale, and 0.95 for the full scale. These values were considered as high^18^.

## Discussion

Nursing is a discipline called to work on the phenomenon of teasing and bullying, as stated by the National Association of School Nurses of the United States^19^. It plays an important and decisive role in the early identification and implementation of bullying prevention strategies by screening the cases with this kind of problem and, therefore, helping to promote the health of students and their families. 

Scientific literature describes several instruments used in research, but few with validity and reliability. For this reason, the cross-cultural adaptation of a nursing measurement instrument was carried out, which measures the frequency of teasing and how much it bothers the student who receives it, and an appropriate methodological quality has been demonstrated^20^ ^-^ ^22^. 

In the process of cross-cultural adaptation of CATS, a scientific and suggested methodology was used to ensure the comparability between the results of Colombian students and those of previous versions^14^ ^,^ ^22^.

The qualitative strategy with the focal groups was fundamental to achieve, among the Colombian students aged 8 to 10 years, with different socioeconomic status and type of educational institution, the understandability of CATS by older students. For this reason, the age interval for application of the instrument was enlarged, ranging from 8 to 15 years in the present study.

It is important to mention that the experts qualified three items of the face validity as unclear or with inappropriate wording; however, these items were clearly understood by students. The above refers to the value that the participation of a group of students similar to the group that will be investigated has, providing validity to the results and compliance with the requirements for an ethical research with children, avoiding biases due to the perspective of adults.

The validity by experts provided a perspective from the point of view of a researcher with scientific knowledge, and from a professional with practical care experience and ability with students, which has contributed significantly considering different points of view and lines of thought. This process has been shown to be important in achieving understandability and cultural coherence in the search for semantic and content equivalence^23^ ^-^ ^24^.

The suggested adjustments were related to the wording of the items and to the observations that were analyzed from the students’ point of view, as an interface between two visions. The importance of the participation of these groups for the validation of scales is confirmed by references in the literature^25^.

The psychometric tests used in this study show the high level of consistency of the results of the Child Adolescent Teasing Scale (CATS) due to its reliability. The interpretation of the results of the two subscales showed that they were classified as high, indicating that the assessment provided by this scale is reliable. In terms of validity, the Kappa coefficient for the semantic equivalence, clarity, consistency and relevance ranged from 0.61 to 0.80, indicating a substantial agreement, and the adequacy showed a moderate agreement, ranging from 0.41 to 0.60, which allowed to conclude that the proportion of times that the evaluators agreed was high^26^ ^-^ ^27^.

As limitations, some authors mention the need to start from the official translation of the original version. For this, the Spanish version of the instrument provided by the author was submitted to different validity and adaptation processes, such as back-translation, assessment by an expert nurse, validity by students and validity by experts. In addition, it was compared with the original English version.

Although the objective of this study was to carry out a qualitative assessment in terms of understandability of the items by representatives of the same population to which CATS would be applied, the Cronbach’s alpha was calculated to achieve reliability, clarifying that due to the small sample size, additional studies could support the psychometric properties of CATS.

Regarding the number of subjects in the pilot sample, there are different opinions, some suggest 30 to 40 people^14^, or 5 to 10 people^22^, because the most important is to achieve understandability on: questions and answers, format of the applied instrument, or problems of the questionnaire^28^. This study was carried out with 19 students, a group that can be an important sample to check the clarity, feasibility and practicality of the instrument. However, further publications will present results on its application in a larger sample of students.

With regard to the phenomenon of school bullying, so widespread, health professionals are advised to perform an interdisciplinary work, since the ultimate goal is the health of students; and the implementation of actions to identify children at risk of bullying, who require care in relation to their health. 

## Conclusion

The cross-cultural adaptation of the Nursing Instrument Child Adolescent Teasing Scale (CATS) for its application in Colombian students aged 8 to 15 was carried out. This version was adapted to the Spanish spoken in Colombia, with the participation of students, by means of the focal group strategy, as well as a pilot test. The participants had high and low socioeconomic statuses, and experts in research on bullying also participated in this study. The adapted version showed desirable results in terms of validity and reliability, which make it possible to point out CATS as a valid and reliable instrument to be applied among students with the aim of identifying the frequency and distress caused by bullying, and it is available for use in future investigations. 
